# Temporal changes in symptomatic intracranial arterial disease: a longitudinal high-resolution vessel wall imaging study

**DOI:** 10.3389/fneur.2025.1583857

**Published:** 2025-06-16

**Authors:** Dong-Wan Kang, Jonguk Kim, Do Yeon Kim, Sung Hyun Baik, Cheolkyu Jung, Bijoy K. Menon, Jae W. Song, Moon-Ku Han, Hee-Joon Bae, Beom Joon Kim

**Affiliations:** ^1^Department of Neurology, Seoul National University College of Medicine, Seoul National University Bundang Hospital, Seongnam, Republic of Korea; ^2^Department of Neurosurgery, Seoul National University Bundang Hospital, Seongnam, Republic of Korea; ^3^Department of Neurology, Inha University Hospital, Incheon, Republic of Korea; ^4^Department of Public Health, Seoul National University Bundang Hospital, Seongnam, Republic of Korea; ^5^Department of Neurology, Gyeonggi Provincial Medical Center, Icheon Hospital, Icheon, Republic of Korea; ^6^Department of Radiology, Seoul National University Bundang Hospital, Seongnam, Republic of Korea; ^7^Department of Clinical Neurosciences, Cumming School of Medicine, University of Calgary, Calgary, AB, Canada; ^8^Department of Radiology, Hospital of the University of Pennsylvania, Philadelphia, PA, United States

**Keywords:** intracranial arterial disease, ischemic stroke, magnetic resonance imaging, high-resolution vessel wall imaging, follow-up MRI

## Abstract

**Introduction:**

The temporal dynamics of the vessel wall in intracranial arterial disease (ICAD) may differ depending on the etiology. We investigated temporal changes in narrowed intracranial arteries after ischemic stroke using serial high-resolution vessel wall imaging (HR-VWI).

**Methods:**

We retrospectively recruited patients with ICAD-related ischemic stroke who underwent two or more HR-VWI scans. The lumen area (LA), total vessel area (TVA), and enhancing area (EA) of the narrowest part of the culprit lesion were manually segmented. Degree of stenosis was estimated as [1-LA/TVA] × 100(%), the enhancing proportion as EA/TVA × 100(%), and enhancement ratio as (T1GD_lesion_/T1GD_ref_)/(T1_lesion_/T1_ref_). Linear mixed models were used to investigate temporal changes in these parameters and whether such changes differed by etiologies.

**Results:**

Of a total of 208 patients, ICAD-related stroke was caused by atherosclerosis (69%), arterial dissection (24%), vasculitis (3%), moyamoya disease (1%), and other (2%). The median follow-up was 319 [IQR, 125–409] days. HR-VWI imaging parameters, namely, degree of stenosis, enhancing proportion, and enhancement ratio showed a trend to decrease over time. Patients with intracranial dissection as a cause of intracranial narrowing showed a faster reduction in degree of stenosis and enhancing proportion vs. when such narrowing was identified as due to atherosclerosis (*β* [95% CI], −0.59%[−0.80% ~ −0.38%] and −0.81%[−1.23% ~ −0.39%], respectively, both *p* < 0.01). The enhancement ratio did not change over time in dissection, while it decreased in atherosclerosis (−0.01 [−0.02 ~ 0], *p* = 0.04).

**Conclusion:**

Intracranial vessel narrowing in patients with ischemic stroke changes over time with different stroke etiologies having their own unique temporal patterns.

## Introduction

Numerous patients experiencing ischemic stroke present with stenosis in the ipsilesional intracranial arteries. This imaging observation indicates intracranial arterial disease (ICAD), which is frequently implicated in the risk of recurrent strokes (1–3). ICAD may stem from atherosclerosis, while it may also manifest as non-atherosclerotic conditions including arterial dissections or vasculitis (4). Distinguishing between these diverse etiologies remains a formidable clinical challenge.

Conventional imaging modalities, such as time-of-flight (TOF) MR angiography or digital subtraction angiography (DSA), predominantly visualize the vascular lumen and are thus insufficient for conclusively identifying the underlying etiology of ICAD, which originates from the vascular walls. In contrast, high-resolution vessel wall imaging (HR-VWI) provides sub-millimeter spatial resolution capable of elucidating reveal vessel wall abnormalities (5). It enables the visualization of distinctive pathologic features associated with various conditions such as intracranial arterial dissection, moyamoya disease, vasculitis, and atherosclerotic plaque (6). Nevertheless, differentiation among these etiologies remain limited due to overlapping radiologic features observed even with HR-VWI (7, 8).

Understanding the unique interplay between pathological changes and natural healing processes in vascular injuries is crucial for interpreting temporal changes in ICAD. Given the paucity of targeted research in this area (9–11), we conducted a detailed analysis of serial HR-VWI data from over 200 patients with ischemic strokes attributable to ICAD. Our focus was on quantifying temporal changes in wall morphology, specifically evaluating the degree of stenosis and the extent of wall enhancement at the culprit segment. This research aims to deepen our understanding of the pathophysiological mechanisms governing ICAD and to discern whether distinct temporal changes might be indicative of specific etiologies.

## Methods

### Study population

**Figure 1:**
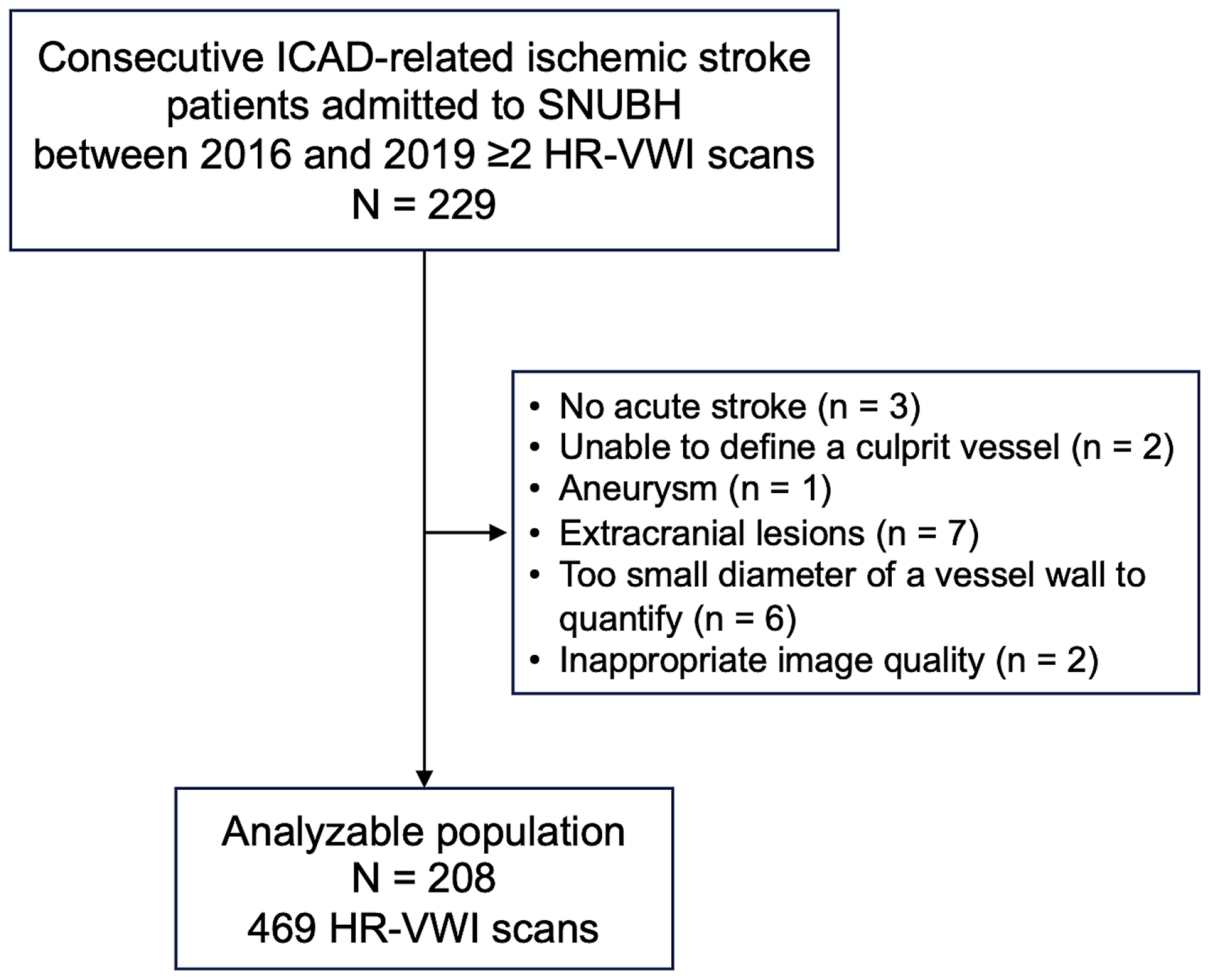
Study profile.

Study subjects were retrospectively identified from a prospective registry of consecutive patients presenting with ICAD-related acute ischemic stroke at the Cerebrovascular Center of the Seoul National University Bundang Hospital, over a period from June 2016 to June 2019 (12). Eligibility for inclusion required patients to have undergone at least 2 HR-VWI scans during their admission and follow-up periods to assess disease progression and response to therapeutic interventions. Exclusion criteria included patients whose final diagnosis did not confirm stroke, those in whom the culprit vessel could not be definitively identified, and cases where the vessel was too diminutive for accurate quantitative analysis (Figure 1).

Stroke management adhered to prevailing clinical guidelines, with HR-VWI scans integrated into routine clinical assessments for ICAD patients. The local institutional review boards approved the study with a waiver of consent (No. B-2102-667-103). The data that support the findings of this study are available from the corresponding author upon reasonable request.

### Clinical characteristics

Baseline demographic and clinical information was retrieved from the prospective stroke registry, encompassing sex, stroke history, and cardiovascular risk factors, such as hypertension, diabetes, dyslipidemia, smoking, and atrial fibrillation. Culprit lesions were defined as the most distal relevant intracranial artery that could account for all observed stroke lesions. These were identified using the initial CT or MR angiographies alongside the HR-VWI scan. The etiology of ICAD was established through a comprehensive clinical and imaging evaluation conducted during the index stroke admission. Clinical evaluation, performed by experienced stroke neurologists, included an assessment of age, associated vascular risks and laboratory tests. Imaging assessments were carried out by experienced neuroradiologists, who performed a detailed review of all available imaging data, including HR-VWI.

ICAD was classified as atherosclerotic when HR-VWI showed typical findings of iso- or hyperdense T1/proton density lesions indicative of a lipid core or high signal lesions on non-contrast T1-weighted imaging suggestive of intraplaque hemorrhage, paired with a corresponding clinical profile (13). Intracranial arterial dissections were diagnosed in the presence of intramural hematoma, double lumen or intimal flap were identified on the HR-VWI or DSA, often accompanied by a sudden severe headache at stroke onset (6). Moyamoya disease (MMD) was diagnosed according to the 2021 diagnostic criteria, which require imaging evidence of stenosis or occlusion at the terminal portion of the intracranial internal carotid artery (ICA) or the proximal portion of the anterior and/or middle cerebral artery (MCA), along with abnormal vascular networks near the lesion and bilateral reduction in the outer diameter of the lesions (14). To confirm the diagnosis, other etiologies such as autoimmune diseases and meningitis were excluded. Notably, HR-VWI revealed characteristic findings of concentric wall thickening with negative remodeling (7, 14, 15). Vasculitis was suspected when HR-VWI findings were suggestive of the inflammatory vasculopathy, characterized by smooth, homogeneous, strong concentric mural enhancement in the intracranial arteries (16–18). The diagnosis was established after confirming neuroinflammation at cerebrospinal fluid and excluding other vasculopathies, including fibromuscular dysplasia, moyamoya disease (MMD), and reversible cerebral vasoconstriction syndrome (RCVS). Vasculitis included both primary angiitis of the central nervous system and secondary vasculitis with identifiable causes such as infection, systemic vasculitis, or malignancy (19).

### HR-VWI protocol

All patients underwent HR-VWI on a 3.0-T MRI scanner (Philips Achieva or Ingenia; Philips Healthcare, Best, The Netherlands) with 8-channel or 32-channel head coils. The imaging protocol was standardized across the cohort over the study period, incorporating T1-weighted image (T1-WI), proton density (PD) images, and gadolinium-enhanced T1 images (T1-Gd) with concurrent TOF MR angiography. Blood signal suppression was achieved using improved motion-sensitized driven-equilibrium (iMSDE) in T1-WI, PD, and T1-Gd sequences. Other common image parameters included a field of view of 180 × 180 mm, matrix of 300 × 300, flip angle of 90 degrees, and a spatial resolution of 0.6 × 0.6 × 0.6 mm^3^. Repetition time/echo time was 570/37 m/sec for T1-WI, 2000/32 m/sec for PD, and 570/37 for T1-Gd. The MR imaging protocol, including both stroke and HR-VWI sequences, took approximately 40 min. MR protocol details are further provided in Supplementary Table 1.

### Image quantification

Image analysis on HR-VWI focused on the most stenotic segment of the culprit lesion and analyzed the vessel’s perpendicular section (20). Manual segmentation and quantification of the lumen area (LA), total vessel area (TVA), and enhancing area (EA) were conducted using ITK-SNAP 4.1 (21). The LA was delineated from the T1-weighted image, while the TVA was measured using PD images at the corresponding plane. The EA was segmented and analyzed at the same most stenotic site on the T1GD image. The EA was quantified into the number of voxels, and its signal intensity was normalized against adjacent normal brain parenchyma by using a manual standard of 15 mm^2^, as reported previously (20, 22).

The degree of stenosis was calculated as (1-LA/TVA) × 100(%), and the enhancing proportion was defined as EA/TVA × 100(%). The enhancement ratio of the enhancing lesion was quantified as (T1GD_lesion_/T1GD_ref_)/(T1_lesion_/T1_ref_).

Blinded to clinical data, three board-certified vascular neurologists (DWK) and interventional neurologists (JK and DYK) with over 5 years of clinical practice independently evaluated HR-VWI scans. A consensus on the segmentation was reached after reviewing and annotating the first 50 cases. The analysis dataset was constructed through independent measurement by three raters, and acceptable inter-rater agreement was documented. Intraclass correlation coefficients for the degree of stenosis, enhancement ratio, and enhancing the proportion of DWK and JK were 0.89, 0.91, and 0.62, respectively. Those of DYK and JK were 0.87, 0.78, and 0.53, respectively. Discrepancies among the raters were resolved through discussions with the senior authors who had 15 years of clinical practice (BJK).

### Statistical analysis

Descriptive statistics were used to summarize the demographic and clinical data. Baseline characteristics were summarized as means ± standard deviations, medians [interquartile ranges], and frequencies (percentages), as deemed appropriate. The quantified imaging parameters were analyzed based on linear mixed-effects regression models with random effects of intercept and slope models to accommodate the hierarchical structure of the data. These models were implemented using the *lme4* package in R. Three progressive models were constructed to explore the effect of various predictors on the imaging outcomes. Model 1 included the fixed effects of time, age, and sex. Model 2 extended model 1 by incorporating ICAD etiologies and their interaction with time. Model 3 further included variables for hypertension, diabetes, and dyslipidemia. All statistical tests were two-tailed, with significance levels at *p* < 0.05. Statistical computations were performed using R, version 4.3.2 (R Foundation for Statistical Computing).

## Results

### Patient characteristics

Of the initial cohort of 229 patients, 21 were excluded from the final analysis for the following reasons: presence of an aneurysm (*n* = 1), extracranial lesions (*n* = 7), absence of acute stroke (*n* = 3), indeterminate culprit vessel (*n* = 2), vessel wall diameter too small for quantitative analysis (*n* = 6), and suboptimal image quality (*n* = 2). Consequently, the study included 208 patients who had experienced acute ischemic stroke and underwent at least 2 HR-VWI scans post-index event. The demographic profile comprised 121 males (58%) with an average age of 57 ± 14 years.

Etiological classification based on clinical and imaging evaluations identified atherosclerosis in 144 cases (69%), followed by arterial dissection in 49 cases (24%). Other identified etiologies included Moyamoya disease in 3 cases (1%), vasculitis in 7 cases (3%), and various other vasculopathies in 5 cases (2.4%)—specifically, antiphospholipid antibody syndrome, cerebral autosomal dominant arteriopathy with subcortical infarcts and leukoencephalopathy (CADASIL), fibromuscular dysplasia, RCVS, and one undetermined case. Analysis of lesion distribution showed that anterior circulation was involved in 132 cases (63%), while posterior circulation was affected in 76 cases (37%) (Table 1 and Supplementary Figure 1). A more detailed description of baseline characteristics stratified by etiology is provided in Supplementary Table 2. The dataset encompassed 469 HR-VWI scans, averaging 2.3 scans per patient with a median inter-scan interval of 319 [IQR, 125–409] days (Supplementary Figure 2).

**Table 1:** Baseline characteristics of the enrolled patients.

Patient characteristics	Total (*N* = 208)
Male sex	121 (58.2%)
Age	56.7 ± 14.3
Total MRI follow-up time (days)	319 [125, 409]
Onset-to-HR-VWI (days)	4 [2, 8]
Hypertension	113 (54.3%)
Diabetes	56 (26.9%)
Dyslipidemia	59 (28.4%)
Smoking	79 (38.0%)
Atrial fibrillation	6 (2.9%)
History of stroke	23 (11.1%)
Coronary heart disease	9 (4.3%)
Medication before stroke	
Antiplatelets	62 (29.8%)
Anticoagulants	1 (0.5%)
Etiology	
Atherosclerosis	144 (69.2%)
Dissection	49 (23.6%)
Moyamoya disease	3 (1.4%)
Vasculitis	7 (3.4%)
Others	5 (2.4%)
Culprit vessel	
dICA	12 (5.8%)
ACA	8 (3.8%)
MCA	112 (53.8%)
BA	17 (8.2%)
VA	41 (19.7%)
PCA	2 (1.0%)
PICA	15 (7.2%)
Posterior choroidal artery	1 (0.5%)

HR-VWI, high-resolution vessel wall imaging; dICA, distal internal carotid artery; ACA, anterior cerebral artery; MCA, middle cerebral artery; BA, basilar artery; VA, vertebral artery; PCA, posterior cerebral artery; PICA, posterior inferior cerebellar artery.

### Distribution of quantified HR-VWI image parameters

In the patients included in this study, the baseline HR-VWI scans revealed a median degree of stenosis at 77.1% [IQR, 56.0–87.6%], a median enhancement ratio of 2.02 [IQR, 1.50–2.59], and a median enhancing proportion of 45.8% [IQR, 29.0–71.6%]. No significant correlations were observed among these image parameters (Supplementary Figure 3). Detailed distributions of these image parameters across different ICAD etiologies are presented in Supplementary Table 3.

### Temporal change of MRI parameters over time

**Figure 2:**
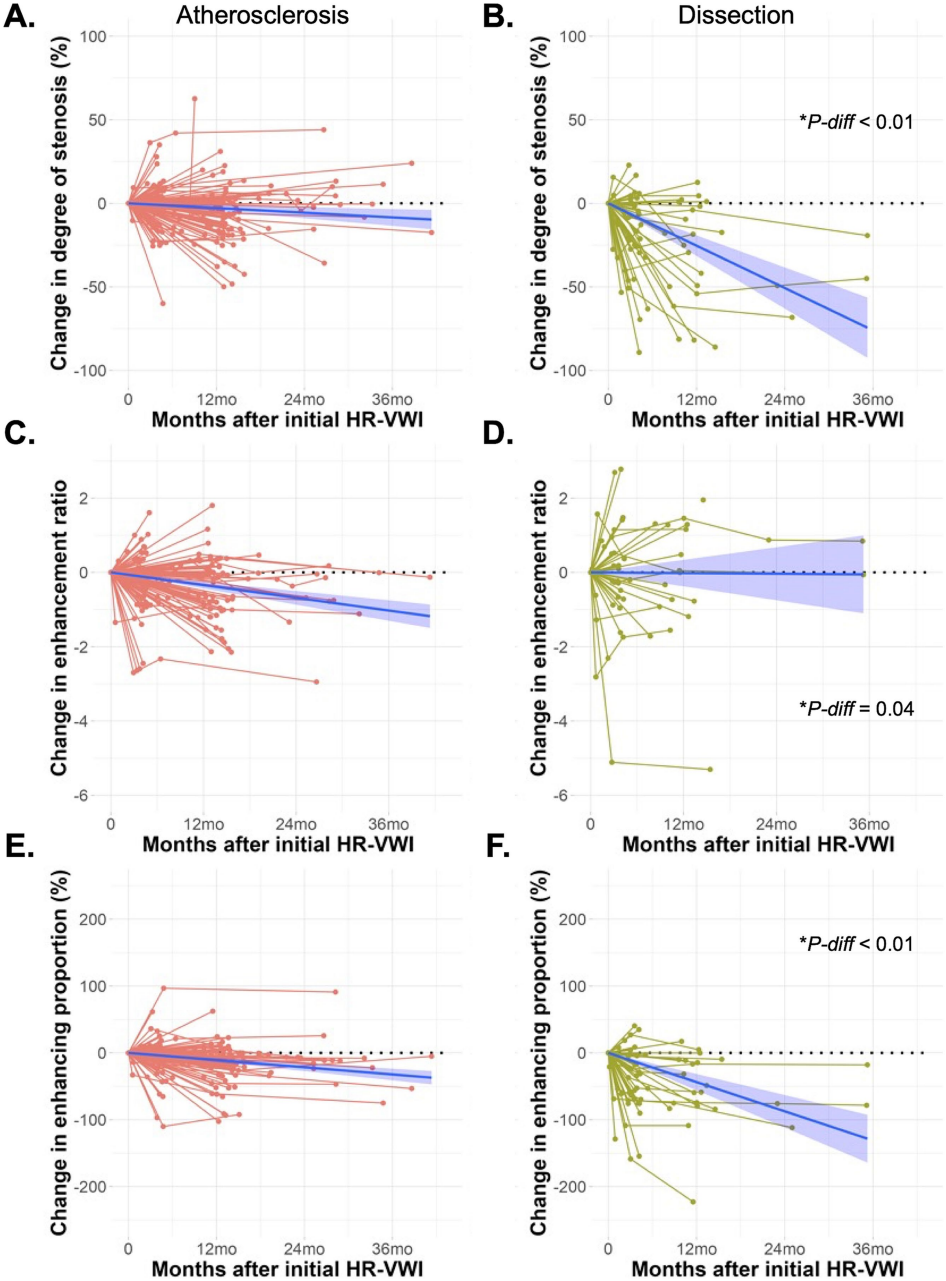
Graphs showing the change in degree of stenosis **(A,B)**, enhancement ratio **(C,D)**, and enhancing proportion **(E,F)** over time for patients with atherosclerosis and arterial dissection. Blue lines are linear regression lines with 95% confidence intervals. *P-diff* indicates *p* values for the difference in MRI parameters between the atherosclerosis and arterial dissection groups over time, derived from linear mixed effects models.

**Figure 3:**
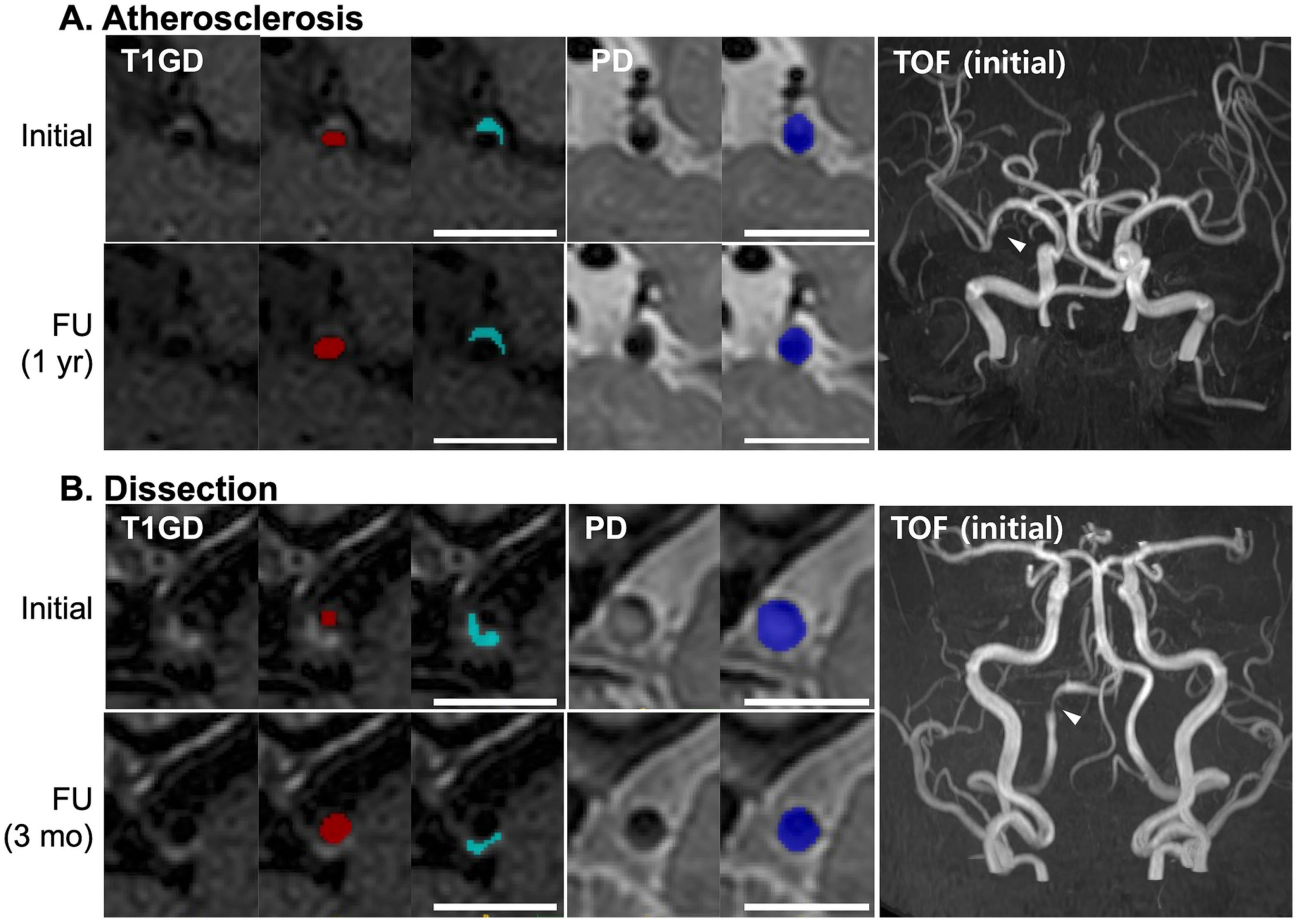
Representative images of the culprit vessels in patients with atherosclerosis **(A)** and arterial dissection **(B)**. Initial and follow-up HR-VWI scans are shown. Lumen area (red), enhancing area (light blue), and total vessel area (blue) are manually segmented. Scale bars, 1 cm.

A longitudinal analysis of the imaging parameters derived from HR-VWI scans indicated a general decreasing trend throughout the follow-up period. Specifically, linear regression analyses demonstrated decreases of 0.17% ± 0.03% in the degree of stenosis, 0.008 ± 0.001 in the enhancement ratio, and 0.39% ± 0.06% in the enhancing proportion per 10-day interval. Notably, these trends varied significantly by ICAD etiology (Figure 2). In the arterial dissection group, the degree of stenosis and the enhancing proportion showed markedly steeper declines compared to those in the atherosclerotic group: 0.70% ± 0.09% versus 0.08% ± 0.02% for the degree of stenosis and 1.33% ± 0.20% versus 0.30% ± 0.04% for the enhancing proportion, respectively (both P-for-difference <0.01). Conversely, the decrease in the enhancing ratio was less pronounced in the arterial dissection group compared to the atherosclerotic group, 0.0005 ± 0.005 versus 0.01 ± 0.001 per 10 days (P-for-difference <0.01). Representative cases illustrating these findings are depicted in Figure 3.

### Temporal dynamics of HR-VWI image parameters by ICAD etiology

In the context of differing ICAD etiologies, the arterial dissection group, when compared to the atherosclerosis group, exhibited a notable reduction in both the degree of stenosis and enhancing proportion over time, as evidenced by linear mixed effects regression models; *β* coefficients were −0.59% (95% confidence intervals [CI] −0.80% ~ −0.38%) and −0.81% (95% CI −1.23% ~ −0.39%), respectively, both achieving statistical significance (*p*-value <0.01, Table 2). In contrast, the enhancement ratio remained unchanged over time in dissection but decreased in atherosclerosis (−0.01 [95% CI −0.02 ~ 0], p-value = 0.04, Table 2 and Supplementary Table 6). Additionally, the moyamoya disease group demonstrated a significant increase in the enhancing proportion over time (2.8% [95% CI 1.343 ~ 4.258], *p*-value <0.01; Table 2).

**Table 2:** Selected β coefficients (β Coeff) and 95% confidence intervals (CI) of the linear mixed effects models for each image parameters.

Variables	Model 1: Time, age, sex, etiology		Model 2: Model 1 + etiology × Time		Model 3: Model 2 + HTN, DM, DL	
	β Coeff (95% CI)	*P*-value	β Coeff (95% CI)	*P*-value	β Coeff (95% CI)	*P*-value
Degree of stenosis (%)						
Time	−0.206 (−0.290 ~ −0.121)	<0.01	−0.111 (−0.203 ~ −0.02)	0.02	−0.11 (−0.201 ~ −0.02)	0.02
Age	0.113 (−0.123 ~ 0.350)	0.35	0.107 (−0.129 ~ 0.343)	0.38	0.093 (−0.148 ~ 0.333)	0.45
Male sex	−1.327 (7.310 ~ 4.656)	0.66	−1.434 (−7.404 ~ 4.537)	0.64	−2.017 (−7.95 ~ 3.917)	0.51
Atherosclerosis × Time			Ref		Ref	
Dissection × Time			−0.593 (−0.806 ~ −0.38)	<0.01	−0.592 (−0.804 ~ −0.381)	<0.01
MMD × Time			0.599 (−0.081 ~ 1.28)	0.09	0.603 (−0.076 ~ 1.282)	0.08
Vasculitis× Time			0.089 (−0.314 ~ 0.492)	0.67	0.091 (−0.31 ~ 0.492)	0.66
Others × Time			−0.171 (−0.737 ~ 0.394)	0.55	−0.159 (−0.719 ~ 0.402)	0.58
Enhancement ratio						
Time	−0.008 (−0.011 ~ −0.005)	<0.01	−0.01 (−0.013 ~ −0.006)	<0.01	−0.01 (−0.013 ~ −0.006)	<0.01
Age	−0.003 (−0.011 ~ 0.004)	0.40	−0.003 (−0.011 ~ 0.004)	0.39	−0.003 (−0.011 ~ 0.005)	0.45
Male sex	−0.233 (−0.431 ~ −0.035)	0.02	−0.238 (−0.437 ~ −0.04)	0.02	−0.258 (−0.456 ~ −0.059)	0.01
Atherosclerosis × Time			Ref		Ref	
Dissection × Time			0.009 (0.001 ~ 0.018)	0.04	0.009 (0 ~ 0.018)	0.04
MMD × Time			0.004 (−0.026 ~ 0.034)	0.79	0.005 (−0.025 ~ 0.035)	0.77
Vasculitis× Time			0.002 (−0.017 ~ 0.021)	0.84	0.001 (−0.018 ~ 0.021)	0.9
Others × Time			−0.001 (−0.021 ~ 0.018)	0.89	−0.002 (−0.022 ~ 0.018)	0.86
Enhancing proportion (%)						
Time	−0.36 (−0.516 ~ −0.203)	<0.01	−0.306 (−0.49 ~ −0.122)	<0.01	−0.291 (−0.474 ~ −0.107)	<0.01
Age	−0.371 (−0.805 ~ 0.063)	0.1	−0.382 (−0.812 ~ 0.048)	0.08	−0.261 (−0.697 ~ 0.175)	0.24
Male sex	−14.031 (−25.03 ~ −3.035)	0.01	−13.93 (−24.86 ~ −3.01)	0.01	−15.63 (−26.41 ~ −4.852)	<0.01
Atherosclerosis × Time			Ref		Ref	
Dissection × Time			−0.803 (−1.227 ~ −0.378)	<0.01	−0.811 (−1.233 ~ −0.389)	<0.01
MMD × Time			2.768 (1.309 ~ 4.226)	<0.01	2.8 (1.343 ~ 4.258)	<0.01
Vasculitis× Time			0.833 (−0.028 ~ 1.693)	0.06	0.776 (−0.085 ~ 1.637)	0.08
Others × Time			−0.096 (−1.157 ~ 0.966)	0.86	−0.026 (−1.081 ~ 1.03)	0.96

MMD, moyamoya disease; HTN, hypertension; DM, diabetes mellitus; DL, dyslipidemia. We used ‘10 days’ as the unit for the time variable. Coefficients for all variables are presented in Supplementary Tables 4, 5, 7.

### Stroke recurrence

A summary of treatments is provided in Supplementary Table 8. Most patients were prescribed antiplatelet agents and statins, and other secondary prevention medications were administered appropriately based on individual comorbidities. Among the included 208 patients, 27 (13.0%) experienced recurrent strokes or transient ischemic attacks (TIA) over a median follow-up period of 335 days (IQR, 107–648). Among those with atherosclerotic stroke, 23 recurrent events (16.0%) were recorded, with 15 cases (65%) originating from the culprit vessel. Notably, no recurrent strokes/TIAs were observed within intracranial arterial dissection. Among the other groups, one of the three patients with MMD experience two recurrent strokes; one of the seven patients with vasculitis had a non-aneurysmal subarachnoid hemorrhage; and one patient with CADASIL experienced two recurrent events, one of which originated from the culprit vessel and another from a different artery.

## Discussion

In this study, we evaluated temporal changes in HR-VWI parameters among 208 patients who underwent serial imaging following an ischemic stroke attributable to ICAD. We observed a general decline over time in the degree of stenosis, enhancing proportion, and enhancement ratio. Notably, these changes were more pronounced in patients with arterial dissection compared to those with atherosclerosis, suggesting differing pathological processes underpin these conditions.

The pathologies associated with ICAD display significant heterogeneity, evident in the varied manifestations of luminal stenosis and contrast enhancement. These differences arise from distinct underlying pathophysiological mechanisms. In atherosclerosis, enhancement typically occurs due to inflammation related to a ruptured cap, fibrous tissues, or ingrowing vasa vasorum (23). This inflammation is particularly pronounced even in the early stages of ischemic stroke when vulnerable plaques are enhanced vividly (24). Conversely, arterial dissections, which can also precipitate stenotic lesions, may show enhancement in the presence of an intraluminal thrombus (25, 26). The evolution of blood products within the intramural hematoma following arterial dissections does not always show the strong T1 shortening typical of methemoglobin in subacute to chronic stages (13), complicating the imaging interpretation. This overlap in imaging features between atherosclerosis and arterial dissections—both displaying stenosis, enhancement and occasionally bright T1 signal – poses a significant diagnostic challenge. Serial HR-VWI may become a critical tool in this context, providing essential diagnostic information as the vascular pathologies evolve distinctly over time, as our results showed. Understanding these temporal dynamics offers valuable insights into the underlying etiology of the disease process, aiding in differentiating between these two common causes of ICAD.

The relatively rapid decrease in both the degree of stenosis and the enhancing proportion observed in patients with arterial dissection can predominantly be attributable to the natural resorption of the mural hematoma. This process reflects a unique aspect of the healing pathway in arterial dissections, differentiating it from the slower, inflammatory-driven changes seen in atherosclerosis. Supporting this observation, prior research has shown that intramural hematomas in arterial dissection typically resolve within 6 months, with significant improvement in most lesions by 12 months (9). Another study also noted that stenosis improved in 30% of patients within a median of 40 days post-dissection (27).

Our observations of gradual improvement in the degree of stenosis, enhancement ratio, and enhancing proportion in patients with atherosclerotic ICAD suggest a possible stabilization of the culprit vessel over time. These findings align with previous studies using serial HR-VWI, which have documented reductions in the degree of stenosis, contrast ratio, surface irregularities, and overall plaque burden within 6 to 12 months following the index stroke event (10, 28, 29). This stabilization likely reflects the healing process of ruptured plaques, which results in a persistent stenotic segment with stable plaque. However, not all findings point towards a uniform improvement in the plaque composition. Some studies, including ours, have observed that certain atherosclerotic lesions may exhibit persistent or even enhancement up to 3 months post-stroke, suggesting ongoing inflammatory processes or other pathological activities within the plaque (11, 30).

The interpretation of HR-VWI features in ICAD remains challenging in the absence of corresponding histologic evidence. Vessel wall enhancement observed in HR-VWI could represent various underlying biological processes, such as increased endothelial permeability leading to contrast leakage, the extent of the vasa vasorum, inflammatory responses, or fibrotic changes. A decrease in the enhancement ratio over time in atherosclerotic ICAD likely signifies the stabilization of an inflamed vulnerable plaque following an acute stroke event. Conversely, the persistent enhancement in arterial dissections may be attributed to the evolution from initial traumatic vessel wall injury to subsequent post-traumatic fibrosis and vascular remodelling (31).

This study, while providing valuable insights into the temporal dynamics of ICAD via HR-VWI, is subject to several limitations that must be clarified. First, this study was conducted at a single center, which follows a standardized HR-VWI protocol. This uniformity implies that our findings may not be directly generalizable to other settings where imaging protocols differ. Secondly, the study included only a small number of patients with MMD, vasculitis, and other etiologies, limiting rigorous analysis to identify trends within these other groups. Thirdly, the statistical models assumed linearity in the temporal changes of MRI parameters for ICAD. However, these changes may follow a nonlinear pattern. Fourthly, we did not define HR-VWI findings as specific vessel wall features such as fibrous cap, vascular calcification, intraplaque hemorrhage, and intramural hematoma. Instead, we identified measurable imaging features that can be followed on serial HR-VWI exams. The identification of such specific histology-related features could potentially lead to a more nuanced understanding of the disease processes in ICAD. Fifthly, the enhancing area was defined based on visual segmentation of hyperintense regions on T1-Gd images, which may have included pre-existing T1 hyperintensities. As such, changes in enhancing area may partly reflect the resolution of baseline T1 hyperintense lesions, such as intramural hematoma, rather than a true decrease in contrast enhancement. Although we attempted to mitigate this by also measuring the enhancement ratio, future studies incorporating voxel-wise comparison between T1 and T1-Gd images may provide deeper insight into the dynamic vessel wall changes in ICAD.

In conclusion, this study has effectively documented temporal changes in HR-VWI parameters among patients with acute ischemic stroke due to ICAD. Notably, alterations were observed in the degree of stenosis, enhancing proportion and enhancement ratio, differed across the clinical diagnosis of atherosclerosis or dissection. These findings underscore the utility of serial HR-VWI in distinguishing between different stroke etiologies, such as intracranial atherosclerosis and arterial dissection, thus aiding in the accurate diagnosis and management of ICAD. Future studies should aim to elucidate the specific factors that contribute to the dynamic temporal changes of ICAD, such as genetic predispositions, the flow dynamics of vascular anatomy, and the role of specific medical interventions. Understanding these factors could lead to more personalized and effective treatment strategies for patients with ICAD.

## Data Availability

The raw data supporting the conclusions of this article will be made available by the authors, without undue reservation.
