# Supplementary material for: Temporal changes in symptomatic intracranial arterial disease: a longitudinal high-resolution vessel wall imaging study
Source: Front Neurol. 2025 Jun 16;16:1583857. doi: 10.3389/fneur.2025.1583857 (PMC12206655; doi:10.3389/fneur.2025.1583857)
Supplement: Supplementary file 1 [file Supplementary_file_1.DOCX]

**Supplementary Material**

**Supplementary Table 1.** Imaging acquisition parameters used in this study.

**Supplementary Table 2.** Baseline characteristics of the enrolled patients stratified by underlying etiology.

**Supplementary Table 3.** Quantified HR-VWI parameters of included subjects at the first scan after the index stroke.

**Supplementary Table 4.** β Coefficients (β Coeff) and 95% confidence intervals (C.I.) of the linear mixed effects models for degree of stenosis (%).

**Supplementary Table 5.** β Coefficients (β Coeff) and 95% confidence intervals (C.I.) of the linear mixed effects models for enhancement ratio.

**Supplementary Table 6.** β Coefficients (β Coeff) and 95% confidence intervals (C.I.) of the linear mixed effects models for enhancement ratio (Dissection group was set as reference).

**Supplementary Table 7.** β Coefficients (β Coeff) and 95% confidence intervals (C.I.) of the linear mixed effects models for enhancing proportion (%).

**Supplementary Table 8.** Summary of treatments after index stroke by etiology.

**Supplementary Figure 1.** The proportion of etiologies in the anterior and posterior circulation vessels.

**Supplementary Figure 2.** Individual follow-up duration of high-resolution vessel wall imaging (HR-VWI) scans.

**Supplementary Figure 3.** Scatter plots showing correlations between image parameters.

|  | **T1-WI** | **PD** | **T1GD** | **TOF-MRA** |
| --- | --- | --- | --- | --- |
| MR sequence | turbo spin echo | turbo spin echo | turbo spin echo | fast field echo |
| Fat suppression | SPIR | No | SPIR | No |
| Black blood technique | iMSDE | iMSDE | iMSDE | NA |
| Dimension | 3D | 3D | 3D | 3D |
| Acquisition orientation | coronal | coronal | coronal | Axial, MIP |
| Repetition time (msec) | 570 | 2000 | 570 | 23 |
| Echo time (msec) | 37 | 32 | 37 | 4 |
| Field of view (mm) | 180 × 180 | 180 × 180 | 180 × 180 | 180 × 200 |
| Flip angle (degree) | 90 | 90 | 90 | 20 |
| Number of excitations | 3 | 2 | 3 | 1 |
| Matrix | 300 × 300 | 300 × 300 | 300 × 300 | 516 × 2360 |
| Spatial resolution (mm) | 0.6 × 0.6 | 0.6x0.6 | 0.6 × 0.6 | 0.6 × 0.6 |
| Slice thickness (mm) | 0.6 | 0.6 | 0.6 | 1.2 |
| Scan time (mm:ss) | 08:05 | 05:38 | 08:05 | 2:21 |
| Interslice gap (mm) |  |  |  | 0.6 |

**Supplementary Table 1. Imaging acquisition parameters used in this study.** T1-WI, T1-weighted image; PD, proton density; T1GD, T1-weighted with gadolinium; TOF, time-of-flight; SPIR, Spectral presaturation with inversion recovery; iMSDE, improved motion-sensitized driven-equilibrium; MIP, maximal intensity projection.

|  | **Total**  **(N = 208)** | **Atherosclerosis**  **(n = 144)** | **Dissection**  **(n = 49)** | **MMD**  **(n = 3)** | **Vasculitis**  **(n = 7)** | **Others**  **(n = 5)** | ***P*-value** |
| --- | --- | --- | --- | --- | --- | --- | --- |
| **Male sex** | 121 (58.2%) | 83 (57.6%) | 32 (65.3%) | 2 (66.7%) | 2 (28.6%) | 2 (40.0%) | 0.363 |
| **Age** | 56.7 ± 14.3 | 61.1 ± 13.4 | 48.5 ± 8.7 | 39.0 ± 6.9 | 38.9 ± 6.6 | 43.8 ± 24.1 | <0.001 |
| **Total MRI follow-up time (days)** | 319  [125, 409] | 362  [130, 419] | 130  [107, 346] | 410  [286, 422] | 355  [335, 414] | 191  [184, 286] | 0.002 |
| **Onset-to-HR-VWI (days)** | 4 [2, 8] | 4 [2, 6] | 8 [ 3, 21] | 4 [4, 5] | 12 [ 6, 23] | 11 [3, 19] | 0.001 |
| **Hypertension** | 113 (54.3%) | 90 (62.5%) | 23 (46.9%) | 0 (0.0%) | 0 (0.0%) | 0 (0.0%) | <0.001 |
| **Diabetes** | 56 (26.9%) | 49 (34.0%) | 4 (8.2%) | 1 (33.3%) | 1 (14.3%) | 1 (20.0%) | 0.01 |
| **Dyslipidemia** | 59 (28.4%) | 50 (34.7%) | 7 (14.3%) | 0 (0.0%) | 1 (14.3%) | 1 (20.0%) | 0.046 |
| **Smoking** | 79 (38.0%) | 55 (38.2%) | 21 (42.9%) | 1 (33.3%) | 2 (28.6%) | 0 (0.0%) | 0.427 |
| **Atrial fibrillation** | 6 (2.9%) | 4 (2.8%) | 2 (4.1%) | 0 (0.0%) | 0 (0.0%) | 0 (0.0%) | 0.951 |
| **History of stroke** | 23 (11.1%) | 17 (11.8%) | 2 (4.1%) | 0 (0.0%) | 2 (28.6%) | 2 (40.0%) | 0.054 |
| **Coronary heart disease** | 9 (4.3%) | 8 (5.6%) | 1 (2.0%) | 0 (0.0%) | 0 (0.0%) | 0 (0.0%) | 0.768 |
| **Medication before stroke** |  |  |  |  |  |  |  |
| Antiplatelets | 62 (29.8%) | 46 (31.9%) | 10 (20.4%) | 0 (0.0%) | 4 (57.1%) | 2 (40.0%) | 0.171 |
| Anticoagulants | 1 (0.5%) | 0 (0.0%) | 1 (2.0%) | 0 (0.0%) | 0 (0.0%) | 0 (0.0%) | 0.515 |
| **Culprit vessel** |  |  |  |  |  |  | <0.001 |
| dICA | 12 (5.8%) | 10 (6.9%) | 1 (2.0%) | 0 (0.0%) | 1 (14.3%) | 0 (0.0%) |  |
| ACA | 8 (3.8%) | 3 (2.1%) | 5 (10.2%) | 0 (0.0%) | 0 (0.0%) | 0 (0.0%) |  |
| MCA | 112 (53.8%) | 99 (68.8%) | 3 (6.1%) | 3 (100.0%) | 3 (42.9%) | 4 (80.0%) |  |
| BA | 17 (8.2%) | 15 (10.4%) | 0 (0.0%) | 0 (0.0%) | 2 (28.6%) | 0 (0.0%) |  |
| VA | 41 (19.7%) | 14 (9.7%) | 27 (55.1%) | 0 (0.0%) | 0 (0.0%) | 0 (0.0%) |  |
| PCA | 2 (1.0%) | 1 (0.7%) | 0 (0.0%) | 0 (0.0%) | 0 (0.0%) | 1 (20.0%) |  |
| PICA | 15 (7.2%) | 2 (1.4%) | 13 (26.5%) | 0 (0.0%) | 0 (0.0%) | 0 (0.0%) |  |
| Posterior choroidal artery | 1 (0.5%) | 0 (0.0%) | 0 (0.0%) | 0 (0.0%) | 1 (14.3%) | 0 (0.0%) |  |

**Supplementary Table 2. Baseline characteristics of the enrolled patients stratified by underlying etiology.** MMD, moyamoya disease; HR-VWI, high-resolution vessel wall imaging; dICA, distal internal carotid artery; ACA, anterior cerebral artery; MCA, middle cerebral artery; BA, basilar artery; VA, vertebral artery; PCA, posterior cerebral artery; PICA, posterior inferior cerebellar artery.

|  | **Total**  **(N = 208)** | **Atherosclerosis**  **(N = 144)** | **Dissection**  **(N = 49)** | **MMD**  **(N = 3)** | **Vasculitis**  **(N = 7)** | **Others**  **(N = 5)** |
| --- | --- | --- | --- | --- | --- | --- |
| Degree of stenosis (%) | 77.1  [56.0, 87.7] | 69.9  [52.8, 85.5] | 87.8  [80.2, 93.4] | 82.1  [49.4, 84.7] | 77.3  [44.9, 85.4] | 83.3  [71.2, 83.6] |
| Enhancement ratio | 2.0  [1.5, 2.6] | 2.0  [1.6, 2.6] | 1.7  [1.2, 2.6] | 2.2  [2.2, 2.3] | 2.0  [1.6, 3.1] | 2.2  [1.8, 2.9] |
| Enhancing proportion (%) | 45.8  [29.0, 71.6] | 38.2  [24.0, 55.3] | 79.6  [58.5, 104] | 51.1  [37.5, 83.9] | 47.6  [42.9, 86.5] | 50.4  [30.2, 129] |

**Supplementary Table 3.** **Quantified HR-VWI parameters of included subjects at the first scan after the index stroke.** MMD, moyamoya disease. Values are median and interquartile range.

|  | **Model 1: Time, age, sex, etiology** | | **Model 2: Model 1 +**  **etiology × Time** | | **Model 3: Model 2 +**  **HTN, DM, DL** | |
| --- | --- | --- | --- | --- | --- | --- |
|  | **β Coeff (95% C.I.)** | ***P*-value** | **β Coeff (95% C.I.)** | ***P*-value** | **β Coeff (95% C.I.)** | ***P*-value** |
| **Degree of stenosis (%)** | | | | | | |
| Time | -0.206 (-0.290 ~ -0.121) | <0.01 | -0.111  (-0.203 ~ -0.02) | 0.02 | -0.11  (-0.201 ~ -0.02) | 0.02 |
| Age | 0.113 (-0.123 ~ 0.350) | 0.35 | 0.107  (-0.129 ~ 0.343) | 0.38 | 0.093  (-0.148 ~ 0.333) | 0.45 |
| Male sex | -1.327 (7.310 ~ 4.656) | 0.66 | -1.434  (-7.404 ~ 4.537) | 0.64 | -2.017  (-7.95 ~ 3.917) | 0.51 |
| Hypertension |  |  |  |  | 3.237  (-3.004 ~ 9.478) | 0.31 |
| Diabetes |  |  |  |  | -7.178  (-14.23 ~ -0.131) | 0.05 |
| Dyslipidemia |  |  |  |  | 6.815  (-0.035 ~ 13.67) | 0.05 |
| Etiology |  |  |  |  |  |  |
| Atherosclerosis |  |  | Ref | | Ref | |
| Dissection | 9.219 (1.903 ~ 16.54) | 0.01 | 12.419  (5.016 ~ 19.82) | <0.01 | 12.35  (4.789 ~ 19.91) | <0.01 |
| MMD | 13.879 (-10.65 ~ 38.40) | 0.27 | 7.898  (-17.46 ~ 33.26) | 0.54 | 11.754  (-13.67 ~ 37.18) | 0.37 |
| Vasculitis | 2.16 (-14.72 ~ 19.04) | 0.80 | 1.251  (-15.92 ~ 18.42) | 0.89 | 2.672  (-14.62 ~ 19.96) | 0.76 |
| Others | 9.688 (-9.543 ~ 28.92) | 0.32 | 10.942  (-8.77 ~ 30.65) | 0.28 | 12.543  (-7.26 ~ 32.35) | 0.22 |
| Etiology × Time |  |  |  |  |  |  |
| Atherosclerosis × Time |  |  | Ref | | Ref | |
| Dissection × Time |  |  | -0.593  (-0.806 ~ -0.38) | <0.01 | -0.592  (-0.804 ~ -0.381) | <0.01 |
| MMD × Time |  |  | 0.599  (-0.081 ~ 1.28) | 0.09 | 0.603  (-0.076 ~ 1.282) | 0.08 |
| Vasculitis× Time |  |  | 0.089  (-0.314 ~ 0.492) | 0.67 | 0.091  (-0.31 ~ 0.492) | 0.66 |
| Others × Time |  |  | -0.171  (-0.737 ~ 0.394) | 0.55 | -0.159  (-0.719 ~ 0.402) | 0.58 |

**Supplementary Table 4. β Coefficients (β Coeff) and 95% confidence intervals (C.I.) of the linear mixed effects models for degree of stenosis (%).** MMD, moyamoya disease; HTN, hypertension; DM, diabetes mellitus; DL, dyslipidemia. We used ‘10 days’ as the unit for the time variable.

|  | **Model 1: Time, age, sex, etiology** | | **Model 2: Model 1 +**  **etiology × Time** | | **Model 3: Model 2 +**  **HTN, DM, DL** | |
| --- | --- | --- | --- | --- | --- | --- |
|  | **β Coeff (95% C.I.)** | ***P*-value** | **β Coeff (95% C.I.)** | ***P*-value** | **β Coeff (95% C.I.)** | ***P*-value** |
| **Enhancement ratio** | | | | | | |
| Time | -0.008  (-0.011 ~ -0.005) | <0.01 | -0.01  (-0.013 ~ -0.006) | <0.01 | -0.01  (-0.013 ~ -0.006) | <0.01 |
| Age | -0.003  (-0.011 ~ 0.004) | 0.40 | -0.003  (-0.011 ~ 0.004) | 0.39 | -0.003  (-0.011 ~ 0.005) | 0.45 |
| Male sex | -0.233  (-0.431 ~ -0.035) | 0.02 | -0.238  (-0.437 ~ -0.04) | 0.02 | -0.258  (-0.456 ~ -0.059) | 0.01 |
| Hypertension |  |  |  |  | 0.006  (-0.203 ~ 0.214) | 0.96 |
| Diabetes |  |  |  |  | -0.176  (-0.413 ~ 0.061) | 0.15 |
| Dyslipidemia |  |  |  |  | 0.229  (0.001 ~ 0.456) | 0.05 |
| Etiology |  |  |  |  |  |  |
| Atherosclerosis |  |  | Ref | | Ref | |
| Dissection | 0.08  (-0.167 ~ 0.328) | 0.52 | -0.049  (-0.324 ~ 0.225) | 0.73 | -0.039  (-0.318 ~ 0.239) | 0.78 |
| MMD | -0.007  (-0.777 ~ 0.763) | 0.99 | -0.089  (-1.039 ~ 0.862) | 0.86 | -0.011  (-0.964 ~ 0.942) | 0.98 |
| Vasculitis | 0.234  (-0.309 ~ 0.778) | 0.40 | 0.188  (-0.451 ~ 0.828) | 0.56 | 0.201  (-0.442 ~ 0.845) | 0.54 |
| Others | -0.013  (-0.628 ~ 0.601) | 0.97 | 0.016  (-0.729 ~ 0.76) | 0.97 | 0.035  (-0.714 ~ 0.784) | 0.93 |
| Etiology × Time |  |  |  |  |  |  |
| Atherosclerosis × Time |  |  | Ref | | Ref | |
| Dissection × Time |  |  | 0.009  (0.001 ~ 0.018) | 0.04 | 0.009  (0 ~ 0.018) | 0.04 |
| MMD × Time |  |  | 0.004  (-0.026 ~ 0.034) | 0.79 | 0.005  (-0.025 ~ 0.035) | 0.77 |
| Vasculitis× Time |  |  | 0.002  (-0.017 ~ 0.021) | 0.84 | 0.001  (-0.018 ~ 0.021) | 0.9 |
| Others × Time |  |  | -0.001  (-0.021 ~ 0.018) | 0.89 | -0.002  (-0.022 ~ 0.018) | 0.86 |

**Supplementary Table 5. β Coefficients (β Coeff) and 95% confidence intervals (C.I.) of the linear mixed effects models for enhancement ratio.** MMD, moyamoya disease; HTN, hypertension; DM, diabetes mellitus; DL, dyslipidemia. We used ‘10 days’ as the unit for the time variable.

|  | **Model 1: Time, age, sex, etiology** | | **Model 2: Model 1 +**  **etiology × Time** | | **Model 3: Model 2 +**  **HTN, DM, DL** | |
| --- | --- | --- | --- | --- | --- | --- |
|  | **β Coeff (95% C.I.)** | ***P*-value** | **β Coeff (95% C.I.)** | ***P*-value** | **β Coeff (95% C.I.)** | ***P*-value** |
| **Enhancement ratio** | | | | | | |
| Time | -0.008  (-0.011 ~ -0.005) | <0.01 | -0.001  (-0.008 ~ 0.007) | 0.89 | -0.001  (-0.008 ~ 0.007) | 0.88 |
| Age | -0.003  (-0.011 ~ 0.004) | 0.4 | -0.003  (-0.011 ~ 0.004) | 0.39 | -0.003  (-0.011 ~ 0.005) | 0.45 |
| Male sex | -0.233  (-0.431 ~ -0.035) | 0.02 | -0.238  (-0.437 ~ -0.04) | 0.02 | -0.258  (-0.456 ~ -0.059) | 0.01 |
| Hypertension |  |  |  |  | 0.006  (-0.203 ~ 0.214) | 0.96 |
| Diabetes |  |  |  |  | -0.176  (-0.413 ~ 0.061) | 0.15 |
| Dyslipidemia |  |  |  |  | 0.229  (0.001 ~ 0.456) | 0.05 |
| Etiology |  |  |  |  |  |  |
| **Dissection** |  |  | **Ref** | | **Ref** | |
| Atherosclerosis | -0.08  (-0.328 ~ 0.167) | 0.52 | 0.049  (-0.225 ~ 0.324) | 0.73 | 0.039  (-0.239 ~ 0.318) | 0.78 |
| MMD | -0.088  (-0.859 ~ 0.684) | 0.82 | -0.039  (-0.992 ~ 0.913) | 0.94 | 0.028  (-0.925 ~ 0.981) | 0.95 |
| Vasculitis | 0.154  (-0.394 ~ 0.702) | 0.58 | 0.238  (-0.406 ~ 0.881) | 0.47 | 0.24  (-0.406 ~ 0.887) | 0.47 |
| Others | -0.094  (-0.716 ~ 0.529) | 0.77 | 0.065  (-0.69 ~ 0.82) | 0.87 | 0.074  (-0.684 ~ 0.831) | 0.85 |
| Etiology × Time |  |  |  |  |  |  |
| **Dissection × Time** |  |  | **Ref** | | **Ref** | |
| Atherosclerosis × Time |  |  | -0.009  (-0.018 ~ -0.001) | 0.04 | -0.009  (-0.018 ~ 0) | 0.04 |
| MMD × Time |  |  | -0.005  (-0.036 ~ 0.026) | 0.75 | -0.004  (-0.035 ~ 0.026) | 0.78 |
| Vasculitis× Time |  |  | -0.007  (-0.028 ~ 0.013) | 0.49 | -0.008  (-0.028 ~ 0.013) | 0.46 |
| Others × Time |  |  | -0.01  (-0.031 ~ 0.01) | 0.33 | -0.011  (-0.032 ~ 0.01) | 0.32 |

**Supplementary Table 6. β Coefficients (β Coeff) and 95% confidence intervals (C.I.) of the linear mixed effects models for enhancement ratio (Dissection group was set as reference).** MMD, moyamoya disease; HTN, hypertension; DM, diabetes mellitus; DL, dyslipidemia. We used ‘10 days’ as the unit for the time variable.

|  | **Model 1: Time, age, sex, etiology** | | **Model 2: Model 1 +**  **etiology × Time** | | **Model 3: Model 2 +**  **HTN, DM, DL** | |
| --- | --- | --- | --- | --- | --- | --- |
|  | **β Coeff (95% C.I.)** | ***P*-value** | **β Coeff (95% C.I.)** | ***P*-value** | **β Coeff (95% C.I.)** | ***P*-value** |
| **Enhancing proportion (%)** | | | | | | |
| Time | -0.36  (-0.516 ~ -0.203) | <0.01 | -0.306  (-0.49 ~ -0.122) | <0.01 | -0.291  (-0.474 ~ -0.107) | <0.01 |
| Age | -0.371  (-0.805 ~ 0.063) | 0.1 | -0.382  (-0.812 ~ 0.048) | 0.08 | -0.261  (-0.697 ~ 0.175) | 0.24 |
| Male sex | -14.031  (-25.03 ~ -3.035) | 0.01 | -13.93  (-24.86 ~ -3.01) | 0.01 | -15.63  (-26.41 ~ -4.852) | <0.01 |
| Hypertension |  |  |  |  | -8.29  (-19.63 ~ 3.049) | 0.15 |
| Diabetes |  |  |  |  | -17.83  (-30.62 ~ -5.041) | 0.01 |
| Dyslipidemia |  |  |  |  | 9.053  (-3.355 ~ 21.46) | 0.15 |
| Etiology |  |  |  |  |  |  |
| Atherosclerosis |  |  | Ref | | Ref | |
| Dissection | 17.34  (3.73 ~ 30.95) | 0.01 | 26.05  (11.12 ~ 40.99) | <0.01 | 23.81  (8.695 ~ 38.92) | <0.01 |
| MMD | 45.90  (1.725 ~ 90.07) | 0.04 | 0.363  (-51.54 ~ 52.27) | 0.99 | 0.082  (-51.72 ~ 51.88) | >0.99 |
| Vasculitis | 62.13  (31.75 ~ 92.51) | <0.01 | 48.15  (13.17 ~ 83.12) | 0.01 | 43.79  (8.758 ~ 78.82) | 0.02 |
| Others | 15.52  (-19.37 ~ 50.42) | 0.38 | 17.14  (-23.08 ~ 57.37) | 0.4 | 12.062  (-28.22 ~ 52.34) | 0.56 |
| Etiology × Time |  |  |  |  |  |  |
| Atherosclerosis × Time |  |  | Ref | | Ref | |
| Dissection × Time |  |  | -0.803  (-1.227 ~ -0.378) | <0.01 | -0.811  (-1.233 ~ -0.389) | <0.01 |
| MMD × Time |  |  | 2.768  (1.309 ~ 4.226) | <0.01 | 2.8  (1.343 ~ 4.258) | <0.01 |
| Vasculitis× Time |  |  | 0.833  (-0.028 ~ 1.693) | 0.06 | 0.776  (-0.085 ~ 1.637) | 0.08 |
| Others × Time |  |  | -0.096  (-1.157 ~ 0.966) | 0.86 | -0.026  (-1.081 ~ 1.03) | 0.96 |

**Supplementary Table 7. β Coefficients (β Coeff) and 95% confidence intervals (C.I.) of the linear mixed effects models for enhancing proportion (%).** MMD, moyamoya disease; HTN, hypertension; DM, diabetes mellitus; DL, dyslipidemia. We used ‘10 days’ as the unit for the time variable.

|  | **Total**  **(N = 208)** | **Atherosclerosis**  **(n = 144)** | **Dissection**  **(n = 49)** | **MMD**  **(n = 3)** | **Vasculitis**  **(n = 7)** | **Others**  **(n = 5)** | ***P*-value** |
| --- | --- | --- | --- | --- | --- | --- | --- |
| **Antiplatelet** | 198 (95.2%) | 138 (95.8%) | 46 (93.9%) | 3 (100.0%) | 7 (100.0%) | 4 (80.0%) | 0.502 |
| **Anticoagulants** | 6  (2.9%) | 4  (2.8%) | 1  (2.0%) | 0  (0.0%) | 0  (0.0%) | 1 (20.0%) | 0.226 |
| **Antihypertensives** | 123 (59.1%) | 94 (65.3%) | 26 (53.1%) | 0  (0.0%) | 1 (14.3%) | 2 (40.0%) | 0.008 |
| **Antidiabetic medications** | 42 (20.2%) | 39 (27.1%) | 1  (2.0%) | 1 (33.3%) | 0  (0.0%) | 1 (20.0%) | 0.003 |
| **Statins** | 181 (87.0%) | 140 (97.2%) | 28 (57.1%) | 3 (100.0%) | 5 (71.4%) | 5 (100.0%) | <0.001 |
| **Immunosuppressive therapy** | 3  (1.4%) | 0  (0.0%) | 0  (0.0%) | 0  (0.0%) | 3 (42.9%) | 0  (0.0%) | <0.001 |

**Supplementary Table 8. Summary of treatments after index stroke by etiology.** MMD, moyamoya disease.


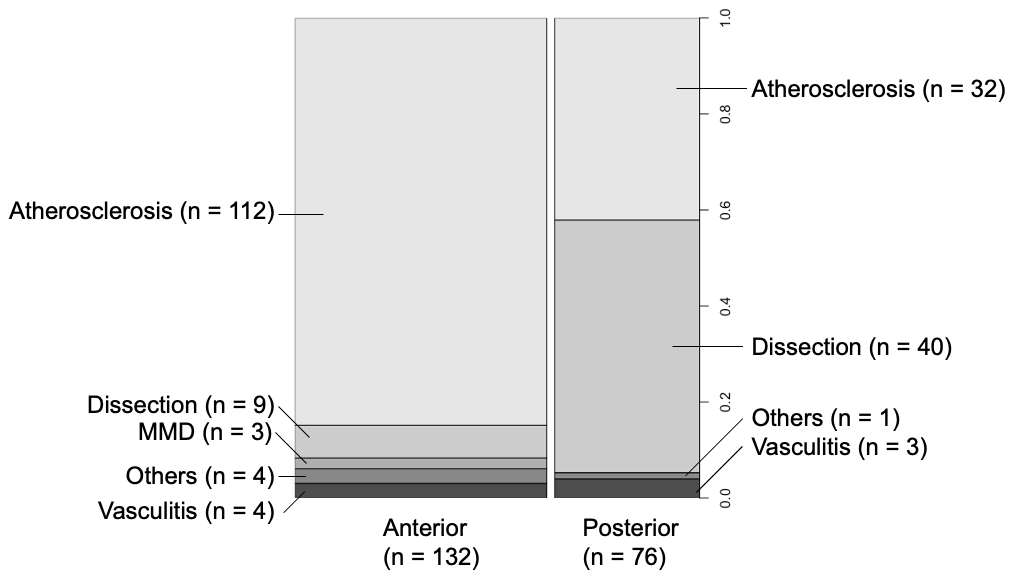


**Supplementary Figure 1. The proportion of etiologies in the anterior and posterior circulation vessels.**


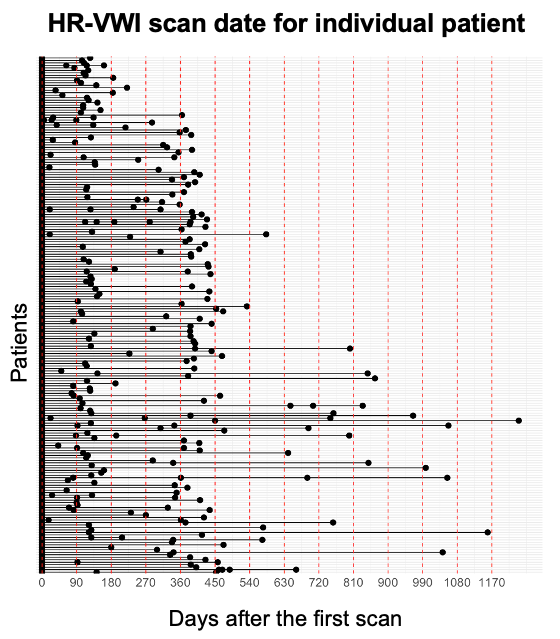


**Supplementary Figure 2. Individual follow-up duration of high-resolution vessel wall imaging (HR-VWI) scans.** The number of HR-VWI scans ranged from two to six (169, 29, 7, 2, and 1, respectively), and the median [IQR] days from the first to the sixth scan were 182 [111, 385], 436 [269, 699], 589 [218, 787], 661 [471, 858], and 386 days, respectively.

**Supplementary Figure 3.** **Scatter plots showing correlations between image parameters.** (A–C) Scatter plots for image parameters of all HR-VWI scans. Correlations between degree of stenosis and enhancing proportion (A), degree of stenosis and enhancement ratio (B), and enhancing proportion and enhancement ratio (C) are shown. (D–F) Scatter plots for image parameters of initial HR-VWI scans. Correlations between degree of stenosis and enhancing proportion (D), degree of stenosis and enhancement ratio (E), and enhancing proportion and enhancement ratio (F) are shown.
